# The ACDSi 2023–24 study protocol: tracking 50 years of physical fitness trends and their determinants in Slovenian children and youth

**DOI:** 10.3389/fpubh.2025.1734046

**Published:** 2026-01-14

**Authors:** Gregor Jurak, Bojan Leskošek, Sara Besal, Kaja Meh, Jerneja Premelč, Petra Golja, Neja Markelj, Urška Kereži, Tatjana Robič Pikel, Katja Zdešar Kotnik, Žan Luca Potočnik, Tjaša Rojko, Nika Bezjak, Vedrana Sember, Marjeta Kovač, Gregor Starc

**Affiliations:** 1 Faculty of Sport, University of Ljubljana, Ljubljana, Slovenia; 2 Biotechnical Faculty, University of Ljubljana, Ljubljana, Slovenia

**Keywords:** anthropometry, health, motor abilities, motor development, physical activity, physical fitness, secular trends, somatic development

## Abstract

The Analysis of Children's Development in Slovenia (ACDSi) is a unique decennial national study that has been monitoring somatic and motor development in Slovenian children and adolescents for more than five decades. It offers unparalleled insight into long-term secular trends in physical fitness and related correlates and determinants. This paper presents the study design and updated methodology of the ACDSi 2023/24 edition, ensuring continuity with previous editions and providing a robust foundation for future comparisons. The ACDSi 2023/24 edition employed a cross-sectional, sentinel-site design, building upon the established methodologies of previous editions. We successfully recruited 3,853 children and adolescents aged 6–18 years, representing 1.35 % of the total population in this age range. The robust research design employed a two-stage cluster sampling technique to ensure national representativeness across various settlement types. Comprehensive data collection included 12 fitness tests, over 25 anthropometric measurements and questionnaires. Surveys were administered online to adolescents (11–18 years) and in paper format to parents of children (6–14 years), capturing information on 24-h movement behavior, commuting habits, sport activity, motivation, self-concept, family environment, socioeconomic background, and nutrition. The methodology establishes a vital database foundation for the subsequent decennial edition, planned for 2033/34. Adherence to this methodology is crucial, distinguishing the ACDSi for its holistic approach to characterizing physical fitness and its determinants. This rigor allows researchers not only to track secular trends in fitness every decade but also to simultaneously observe shifts in the complex determinants affecting fitness trends in children and adolescents. The ACDSi 2023/24 study preserves the methodological continuity of earlier editions by generating harmonized datasets, while simultaneously broadening its scope through the inclusion of additional indicators that capture the influence of evolving social and technological environments on children's and adolescents' lifestyles.

## 1 Introduction

Physical fitness, typically described as the capacity to perform everyday physical tasks without excessive fatigue and with sufficient energy reserves (1), is recognized as a robust predictor of present and future health (2–5). Promoting appropriate fitness levels in childhood and adolescence is thus essential for safeguarding long-term health trajectories at the population level.

Of all components of physical fitness, cardiorespiratory and muscular fitness have demonstrated the most consistent causal relationships with health outcomes. Several studies have linked poor cardiorespiratory and/or muscular fitness in young people to increased risk of developing cardiovascular and cardiometabolic disease risk factors (2–4), cardiovascular disease morbidity and mortality (6–13), psychiatric diseases and suicide (6, 14), and all-cause disability and mortality (6, 7, 9, 10, 13, 15). Moreover, having improved cardiorespiratory and muscular fitness during childhood and adolescence proved to be associated with lower levels of blood pressure (16), better bone health (17, 18), improved immune function (19), better mental health (14), enhanced cognitive and academic performance (20–23), higher neuroelectric activity (24), and larger gray matter and total brain volumes (25).

Given the well-established links between childhood fitness and lifelong health, regular monitoring of secular trends in physical fitness among children and adolescents is essential for understanding how societal and technological changes influence population health. Tracking these trends allows researchers and public health professionals to quickly identify emerging health risks, better inform policy decisions, and efficiently promote physical fitness to maintain or increase a society's productivity and overall well-being.

Secular trends in physical fitness among children and adolescents must be interpreted with caution, as patterns differ by fitness component. The most notable decline has occurred in cardiorespiratory fitness, although this trend appears to have stabilized for some groups after approximately 2012. After a prolonged period of decline, muscular strength has shown modest improvement in recent years, whereas muscle power continues to demonstrate a slight negative trend. Furthermore, speed performance has improved slightly to moderately since 2002, although this positive change has been observed only in specific subgroups (26). In parallel to physical fitness decline, the global, age-standardized prevalence of childhood obesity has tripled between 1990 and 2021 (27). These pervasive trends are largely attributed to altered lifestyles, including increased sedentary behavior and reduced physical activity opportunities, and have led to concomitant rises in childhood obesity rates (24, 28–30). This deterioration in health markers subsequently resulted in increased rates of non-communicable diseases in ever-younger population (28–30). The declines in fitness and the increasing obesity trends were particularly noted during and after the COVID-19 pandemic (31–33).

Adequate access to physical activity and physical fitness resources represents a fundamental equity issue (34). Children and adolescents from less developed regions and, critically, from low-socioeconomic-status (SES) families often display lower physical fitness levels due to multiple interconnected barriers. These barriers include significant financial obstacles to participation in organized sports and limited access to a high-quality, nutritious diet, which collectively fuel lower fitness and higher obesity rates (35, 36). Conversely, children from families with higher SES enjoy more robust financial options for sport and healthy eating and are more likely to harbor a heightened awareness of the effective healthy lifestyle maintenance (36).

Slovenia has a long-standing tradition of monitoring children's physical fitness, offering valuable insight into population-level trends. This effort relies on two complementary data sources: the annually collected SLOfit system data (37) and the more comprehensive, decennial datasets from the Analysis of Children's Development in Slovenia (ACDSi) study (38, 39).

The aim of this paper is to present the methodology of the most recent research edition of the ACDSi 2023/24 study. Building on a reach legacy, this protocol update is critical for sustaining the long-term monitoring of fitness and its determinants and extending the dataset span over 50 years.

### 1.1 A summary of the ACDSi study legacy

The ACDSi 2023/24 edition is the latest iteration of an ongoing, secular trend study, with its roots dating back to 1970–71. In those years, the Faculty of Sport at the University of Ljubljana launched a pioneer study, initially entitled “Comparisons of some motor and morphological parameters in primary schools in Slovenia.” This project has evolved considerably over the subsequent decades. Since its inception, the ACDSi study has been implemented in decennial cycles −1970/71 (40), 1983 (41), 1993/94 (42), 2003/04 (43), 2013/14 (38, 39), and 2023/24—making it one of the longest-running investigations of secular trends in child and adolescent physical fitness and its determinants worldwide.

The original 1970/71 study employed a nationally representative sample, selected through a multi-stage, stratified sampling design (40, 44). Ten research sites were chosen to represent diverse types of residence: from rural to rural-industrial, industrial-rural and industrial and various regions across Slovenia. At each study site, one primary school was selected, and beginning in 1993 an additional school in the capital region was included to ensure appropriate population representation. In 1994, the ACDSi's study scope expanded to include upper secondary school students, aged 15 to 18, across three novel sites. This expansion involved adding six upper secondary school centers, with different educational program specializations. Subsequently, each consequent edition has been spanning over two consecutive years: in the first year, primary school children are being assessed, followed by upper secondary school students in the second year. In each edition, the ACDSi study consistently includes approximately 5,500 children and adolescents aged 7 to 18 years, representing around 2% of the entire population within the age range (Table 1). Notably, reforms in Slovenia's education system introduced an earlier primary school entry age for the 2003 cohort, expanding the sampling frame to include children as young as six. In earlier editions, each age and sex cohort included around 200 participants.

**Table 1:** ACDSi sample in different study editions.

**Year of study**	**Principal investigators**	**Sample age span (years)**	**Total sample size**
1970/71	Šturm, J.	7–14	3,272
1983	Šturm, J., Strel, J.	7–14	3,163
1993	Strel, J., Šturm, J., Kovač, M.	7–14	3,488
1994		15–18	1,620
2003	Strel, J., Kovač, M., Starc, G., Jurak, G.	6–14	4,095
2004		15–18	1,694
2013	Starc, G., Jurak, G., Kovač, M.	6–14	3,796
2014		15–18	1,608
2023	Jurak, G., Starc, G.	6–14	2,721
2024		15–18	1,139

### 1.2 Evolution of the ACDSi study contents

The scope of the ACDSi study has evolved over time. Initially, the focus was on physical fitness and physical anthropology, employing standardized tests prevalent in the former Yugoslavia and Eastern European countries (44–46). In 1993, the inclusion of tests from the Eurofit test battery (47) were incorporated, broadening the study's scope to encompass a more internationally comparable range of fitness tests.

Since its inception, the ACDSi study has progressively expanded its analytical scope to investigate key correlates and determinants of physical fitness and their interrelationships, including somatic development, motor abilities, cognitive functioning, social factors, and environmental influences. This expanded focus allowed for a more comprehensive understanding of physical fitness and factors shaping youth development in any given generation.

Although some data collection methods have varied somewhat across different study editions, the core dataset remained consistent, comprising of:

▪ over 25 anthropometric variables
▪ more than 12 fitness variables
▪ psychological, socioeconomic, and parental attitude data

Unfortunately, the original 1970/71 individual-level data was lost in a fire, limiting comparisons to summary data published in the 1970/71 study report. Table 2 outlines the specific category of variables from each edition, including the updated 2023/24 dataset. Supplementary Table 1 presents detailed dataset information from all editions.

**Table 2:** Overview of observed domains through all ACDSi editions.

**ACDSi study edition**	**1970–71**	**1983**	**1993**	**1994**	**2003**	**2004**	**2013**	**2014**	**2023**	**2024**
Physical fitness tests	•	•	•	•	•	•	•	•	•	•
Anthropometric measurements	•	•	•	•	•	•	•	•	•	•
Physiological indicators		•	•	•	•	•	•	•	•	•
Birth data of a child							•	•	•	•
24-h movement behavior		•	•	•	•	•	•	•	•	•
Commuting to school					•	•	•		•	
Sporting activity		•	•	•	•	•	•	•	•	•
Motivation and self-concept		•	•	•			•	•	•	•
Health status		•	•	•	•	•	•	•	•	•
Diet		•	•	•	•	•	•	•	•	•
Family environment and close friends		•	•		•	•	•	•	•	•
Other child's activities		•	•	•	•	•	•	•	•	•
School perceptions		•	•	•	•	•	•	•	•	•
Socioeconomic environment		•	•	•	•	•	•	•	•	•

### 1.3 ACDSi study data collection details

The Faculty of Sport at the University of Ljubljana leads the administration and implementation of the ACDSi study. The Principal Investigators oversee all phases of the research process, including study design, funding acquisition, coordination with local teams and participating schools, fieldwork organization, training of the measurement team, and data management—encompassing cleaning, analysis, and interpretation.

Preparations for each study edition start at least 1 year in advance. We start by informing the municipality mayors and school principals to ensure their support early on. We then collaborate with physical education teachers in each jurisdiction to appoint school-level coordinators.

These local coordinators work with our core research team to communicate with other teachers and parents, integrate the study into the school's annual teaching plan, collect parental consent and child assent, and coordinate the measurement schedule. Based on completed consent forms, the core research team creates a detailed participant list, each with unique IDs.

To ensure accurate measurements, the core research team recruits and thoroughly trains student investigators in June and September of each study year. During each testing session, a team of between 20 and 25 student investigators and experienced researchers is deployed to a given location. The team is then split into four groups, each comprised of a group lead. The four groups and core responsibilities are:

1. Reception: manages participant identity, coordinates groups' flow between measurement sites, verifies data entry, collects and stores the results sheets, and distributes and collects parent questionnaires.
2. Anthropometric group: conducts anthropometric measurements in a classroom or small gym.
3. Fitness testing group: administers fitness tests in the gym or outdoors.
4. Questionnaire group: oversees the completion of web-based questionnaires in school computer rooms.

The described rigorous organizational procedures have been presented across all data collection phases and ensure consistent and reliable data collection between each of the ACDSi study editions.

## 2 The ACDSi 2023/24 study methodology

### 2.1 Main objectives and areas of interest of the present study

The primary objective of the 2023/24 ACDSi study edition was to assess the current state of somatic and motor development in Slovenian children and adolescents to compare it with data from previous editions of ACDSi study. This involved addressing the following specific research questions:

1. What are the current levels of cardiorespiratory fitness, muscular fitness, body composition, and anthropometric characteristics in Slovenian children and adolescents, and how do these levels vary by age and sex?
2. What are the daily patterns of physical activity, sedentary behavior, and sleep among contemporary Slovenian children and adolescents, and to what extent do these patterns meet existing public health recommendations?
3. Which demographic, behavioral, environmental, and psychosocial factors are associated with physical fitness and 24-h movement behaviors in Slovenian children and adolescents?
4. How has physical fitness in Slovenian children and adolescents changed over the past 50 years, and how do these secular trends compare with recent international data?
5. How have anthropometric characteristics of children and adolescents (e.g., body height, body mass, BMI, body composition) changed over the past 50 years, and how do these trends compare with corresponding international patterns?

A list of more detailed descriptive research goals included:

- What are the socioeconomic characteristics of Slovenian children and adolescents, and how do these characteristics relate to their health, movement behaviors, and physical fitness?
- What are the current somatic characteristics of children and adolescents—body height, body mass, BMI, hip and waist circumferences, skinfold thicknesses, and body fat percentage—and how do these values compare with updated national growth charts?
- How have indicators of physical maturation (e.g., tempo and timing) and body shape (somatochart) changed in Slovenian adolescents over the past decades?
- What are the current levels of cardiorespiratory fitness, muscular fitness, and specific physical fitness test performances in Slovenian children and adolescents, and how do these align with updated national normative charts?
- How have anthropometric characteristics and physical fitness levels of Slovenian children and adolescents changed over the past 10, 20, 30, 40, and 50 years?
- How do these long-term changes compare with secular trends observed in international populations?
- How has the distribution (not only the mean level) of physical fitness performance changed across the population of children and adolescents during the past 50 years?
- How has biological maturation (e.g., changes in body size, body proportions, and maturation timing) in Slovenian adolescents evolved over the past 40 years?
- How have these 24-h movement behaviors patterns changed over the past 10 years?
- What are the current sport participation habits of children and adolescents (frequency, type of sport, organizational form), and how have these changed over the past 10, 20, 30, and 40 years?
- How do children and adolescents perceive their own health, and how has self-perceived health changed over the past 10 years?
- What types of social support (parental support, parental attitudes toward physical education, physical activity of close friends) do Slovenian children and adolescents receive, and how has this support changed over the past 10, 20, and 30 years?
- What are the psychological attitudes toward physical activity—including motives and self-concept—and how have these changed over the past 10, 20, and 30 years?
- What is the current intake of food supplements and energy drinks among children and adolescents, and how has this behavior changed over the past 10 years?

In addition to descriptive research goals, we also established several prediction goals based on existing literature. These were to determine or observe:

- How do socioeconomic characteristics and home-environment attributes relate to adolescents' cardiorespiratory fitness, muscular fitness, body composition, overweight/obesity, and 24-h movement behaviors?
- To what extent does sleep duration and quality predict physical activity levels and sedentary behavior in children and adolescents?
- Is biological maturation a significant predictor of physical fitness components in children and adolescents?
- How does physical fitness in childhood and adolescence predict body composition, health indicators, and self-concept?
- How are health risks in children and adolescents associated with overweight/obesity, different levels and types of physical activity, and cardiorespiratory and muscular fitness
- How are 24-h movement behaviors and physical fitness related to motives for physical activity and self-concept in children and adolescents?
- How do 24-h movement behaviors, sport participation, and physical fitness vary across socioeconomic strata in children and adolescents?

### 2.2 Study design

The ACDSi is a decennial, cross-sectional sentinel-site study conducted since 1970–71, involving 11 primary schools and 10 upper secondary schools. The 2023/24 edition aimed to recruit a total of *n* = 4,200 participants, with a target of *n* = 150 per age-by-sex group (6–18 years). This corresponds to approximately *n* = 2,700 primary school students (6–14 years) and *n* = 1,500 upper secondary school students (15–18 years). This target sample size is lower than in previous editions (*n* = 200 per group), reflecting demographic changes—specifically, the decreasing number of school-aged children in Slovenia—and optimization of study costs. Importantly, power analyses confirmed that the revised sample remains sufficient for all planned statistical comparisons.

No incentives were provided to participants for their participation.

### 2.3 Final sample selection

The ACDSi 2023/24 study employed a two-stage cluster sampling design with schools as primary sampling units and classes as secondary ones. Sample stratification at school level included:

▪ School selection: we obtained the number of classes and students from each school through school coordinators. The number of students recruited from each school was proportional to the size of the settlement. This ratio mirrored the original 1970–71 ACDSi study, and the national population distribution based on settlement types (data from the Statistical Office of the Republic of Slovenia). Maintaining this sample structure ensured the current study's representativeness across different settlement types and sizes was retained.
▪ Class selection: within each selected school, classes were randomly selected to achieve the target sample size. The probability of a class to be selected in a final sample was proportional to number of classes in each grade of each school, size of the classes and estimated response rate (80% in primary schools and 50% in secondary schools). In smaller schools (e.g., the ones with only two classes per grade), this meant one of the two classes was randomly selected, whereas in larger schools, two or three classes were randomly selected from each grade.
▪ Parental consent: all students from selected classes were invited to participate in the study. Parental consent was obtained for all participating students. If enough parental consent were not obtained from a specific school, randomly selected students from additional classes from the same school were invited, until the estimated target sample size was met.

The target sample size was 300 participants per grade, corresponding to 2,700 students across nine primary school grades and 1,200 students across four secondary school grades. The actual sample size (2,721 in primary schools and 1,139 in secondary schools; Table 1) closely matched the planned targets.

**Figure 1:**
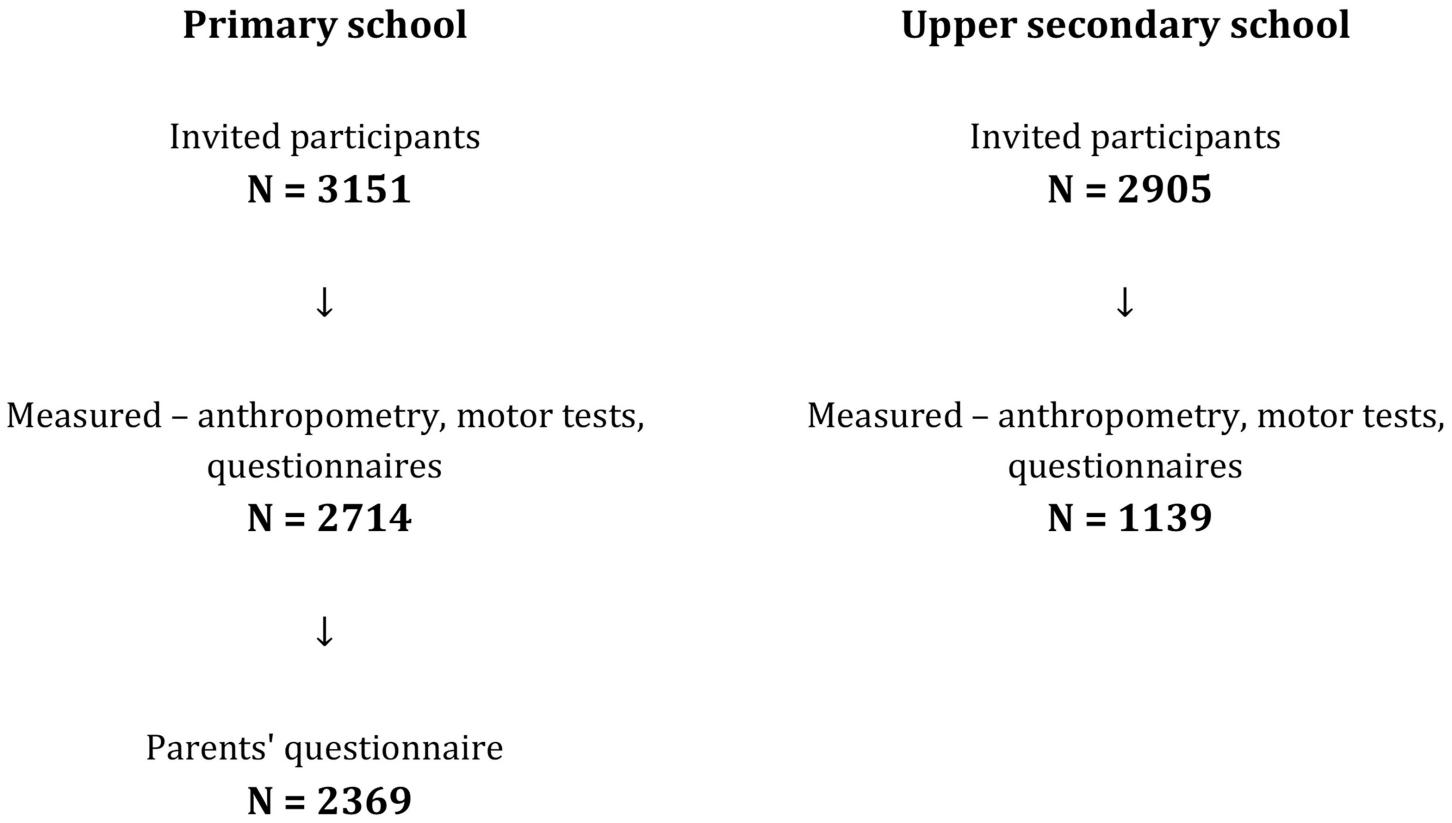
Flow chart of the participants included in the ACDSi 2023/24 study.

By following this sampling strategy, the ACDSi 2023/24 study aimed to obtain a representative sample of Slovenian youth consistent with previous cycles (Figure 1).

The initial target for the ACDSi 2023/24 study was deliberately inflated to *n* = 6,056 participants (3,151 primary school students and 2,905 upper secondary school students) to account for the anticipated non-response and dropout. This upward adjustment was based on historical rates of approximately 20% for primary school and 50% for upper secondary school age groups. The final sample size of participants who successfully completed all fitness testing, anthropometry examinations, and questionnaires was *n* = 3,853 (Table 3; five participants did not provide information on sex and birth date). This corresponds to 63.7% of the initially invited sample and represents 1.35% of the total Slovenian population aged 6–18 years in 2024 (48).

**Table 3:** The final ACDSi 2023/24 study sample, stratified by sex and age.

**Age (years)**	**Males**	**Females**	**Total**
5	25	34	59
6	145	138	283
7	176	137	313
8	162	132	294
9	168	143	311
10	126	123	249
11	169	140	309
12	172	151	323
13	173	146	319
14	161	153	314
15	152	171	323
16	159	161	320
17	120	105	225
18	97	46	143
19	32	18	50
20 and more	5	8	13
Total	2,042	1,806	3,848

Age is truncated to an integer, e.g., 6.0 to 6.9 is 6.

### 2.4 Sampling results

In addition to the school-based measurements, paper questionnaires were distributed to parents of primary school students. Considerable effort was devoted to encouraging active participation, which yielded a satisfactory response rate of 89.6 % of obtained questionnaires.

### 2.5 Main methods of data collection

All tests and measurement protocols used in the ACDSi 2023/24 study have been validated for use in children and adolescents within the targeted age ranges (see Table 4).

**Table 4:** Summary of the data collection undertaken in the ACDSi 2023/24 study.

**Method of data collection**	**Target group**	**Research area**
Physical fitness testing	Children 6–18 years	Cardiorespiratory fitness, muscular fitness, skill-related fitness.
Physical measures	Children 6–18 years	Anthropometry: standard anthropometric procedures, blood pressure, biological maturation.
Web-based questionnaires	Children 11–18 years	Social-economic environment, support of parents for physical activity, close physical active friends, 24-HMB, commuting to school, sporting activity, self-precepted health, prioritizing of school subjects, academic achievements, motives for physical activity and self-concept.
Structured interview	Children 11–18 years	Intake of food supplements and energy drinks.
Pen-and-paper questionnaires	Parents of children 6–14 years	Age, education, body mass and height, birth data of child, commuting to school opinion about the importance of school subjects, social-economic status, siblings.

### 2.6 Testing details

Fitness testing, anthropometric measurements, and questionnaire administration were conducted between September 1 and October 4, 2023, for primary school students, and between September 12 and 27, 2024, for upper secondary students. Measurements were organized between 8:00 AM and 2:00 PM and typically completed within 1 or 2 days per school, depending on the sample size. Every child or adolescent was present for measurements in 1 day.

A trained measurement team composed of student investigators from several faculties of the University of Ljubljana (e.g., Sport, Biotechnical Sciences, Health Sciences, Arts, and Education) conducted all assessments. Three senior researchers acted as field supervisors overseeing the procedures for anthropometry, fitness testing, and questionnaire administration. Any equipment not typically available in school gymnasiums was provided on-site by the core research team. All testing equipment was routinely calibrated every morning before testing began, and throughout the testing period (when necessary). A complete list of the ACDSi 2023/24 research team is acknowledged in the Supplementary material.

Anthropometric measurements were conducted in smaller gyms or classrooms maintained at a temperature of 20 °C−24 °C. Fitness tests were administered in school gyms under similar temperature conditions. Outdoor running tests were conducted only when the temperature exceeded 10 °C, and in the absence of heavy rain or wind. Web-based questionnaires were completed by participants in school computer labs, before or after fitness and anthropometric measurements in the gym, with groups of up to 22 students per session.

Each gym measurement session involved 40–60 participants, divided into two groups: one for anthropometric measurements, and the other for fitness tests. Each session lasted approximately 90 min. Blood pressure was measured for all participants in seated position before any physical exertion took place. A standardized warm-up was led by the research team immediately preceding fitness testing.

All participants aged 11 and older completed a web-based questionnaire in a computer room under the guidance of a responsible investigator. Upon arrival in the computer room, each participant received a unique login ID.

After logging in, students completed the questionnaire, while investigators provided individual assistance when required.

After completion of the on-site student measurements, questionnaires for parents of children aged 6–14 years were distributed. These were to be completed at home and returned to the school coordinators the following day.

All data collected in the ACDSi 2023/24 study are jointly owned by the research team, with the principal investigators responsible for database management. The database, together with detailed measurement protocols, is available to the scientific community upon request for comparative analyses.

A detailed description of all anthropometric measures and fitness test items can be found on the study website in the document Motor_anthropometry_tests.pdf.

#### 2.6.1 Physical fitness tests

Our approach to physical fitness measurements extends beyond the limited scope of laboratory-based measures (49). We incorporated a range of established field-based fitness tests, building upon a robust lineage of data from previous cycles of the ACDSi study. For this reason, physical fitness tests were conducted and scored using SLOfit (37, 42) and EUROFIT protocols (47). Although some tests were adapted for younger children, all protocols have been validated on a sample of Slovenian population, proving their appropriateness for the selected population (44, 45). The measurements in the ACDSi 2023/24 measurements included 12 physical fitness tests in total (Table 5).

**Table 5:** Measured items in ACDSi 2023-24 by research categories.

Physical fitness tests	Anthropometric measurements
• 20-s plate tapping test • Standing broad jump • 20-s sit-ups test • 60-s sit-up test • Polygon backwards test • Sit and reach test • Shoulder circumduction test • 30-s drumming test • Flamingo balance test • Arm hang test • Handgrip strength test • 30-m sprint test • 60-m sprint test • 20-m shuttle run test	•Self-reported body height • Self-reported body mass • Body height • Body mass • Sitting height • Shoulder breadth (biacromial) • Pelvis breadth (biiliocristal and bispinal) • Ankle breadth (bimalleolar) • Femoral breadth (biepicondylar femur) • Elbow breadth (biepicondylar humerus) • Wrist breadth (lateral-medial stylion) • Arm length (acromion-dactylion) • Leg length (iliospinale-basion) • Foot length (acropodion-pterion) • Flat foot (Clarke angle) • Triceps skinfold • Biceps skinfold • Suprailiac skinfold • Supraspinal skinfold • Subscapular skinfold • Anterior thigh skinfold • Medial calf skinfold • Abdominal skinfold • Forearm circumference • Mid-upper arm circumference relaxed • Mid-upper arm circumference flexed • Calf circumference (the widest part of calf) • Gluteal thigh circumference • Mid-thigh circumference • Waist circumference (the narrowest part between ribs and iliac crest) • Hip circumference (the level of the most prominent buttocks)
24-HMB • Moderate physical activity • Vigorous physical activity • Strength exercise • Bone-health exercise • Screen time • Reading time • Sleep time • Quality of sleep • Playing instrument • Playground time	
Commuting to school • Mode of commuting to school • Distance from place of residence to school • Reasons to use mode of commuting to school	
Sporting activity • Current volume • Past volume • Organizational form • Sport disciplines	
Motivation and self-concept • Intrinsic motivation • Self-determined extrinsic motivation • Non-self-determined extrinsic motivation • Amotivation • Physical Ability • Physical Appearance • Peer Relations • Parent Relations • Reading • Mathematics • General-School • General-Self
	Physiological indicators • Age at menarche (girls only) • Blood pressure
	Birth data of a child • Gestational age (weeks) • Birth mass • Birth length • Duration of breastfeeding
	Energy drinks intake • Energy drinks consumption
Socioeconomic environment • Education of parents • Perceived financial well-being • Family lifestyle indicator	Health status • Health index score • General health
Family environment and close friends • Type of the family • Age of parents • Parents self-reported body mass and hight • Parents' support for physical activity • Siblings • Close friends	School perception • Homework • The most important school subject for life • The most and the least favorable school subject • Perceived school related stress level • Math grade

Participants performed all the fitness tests in shorts and t-shirts, either shod or barefoot, depending on the test requirements. Prior to each test, a measurement team member explained and demonstrated the execution of the test. During testing, participants did not receive verbal encouragement. If a test was not performed correctly, the participant was permitted to repeat the attempt. Less physically demanding tests were repeated twice. The 20-s sit-ups and 30-meter run were performed by participants aged 5–11 year only. The 20-s tapping test was administered using an electronic arm-plate (Elan, Begunje, Slovenia), and handgrip strength was assessed with a Jamar hydraulic hand dynamometer (Bolingbrook, IL, USA).

#### 2.6.2 Anthropometric measurements

All but three anthropometric measurements, were conducted in accordance with the standards of the International Society for the Advancement of Kinanthropometry (ISAK) (50). The three exceptions: height, sitting height, and flexed arm circumference—were measured following the protocol of Lohman (51) to ensure continuity with data collected in previous decades. In contrast to ISAK standards, Lohman's (51) protocols specify that body height and sitting height are to be measured without stretching. Flexed arm circumference was assessed at the midpoint between the anthropometric landmarks' acromion and radiale, rather than at the point of maximal circumference of the contracted biceps brachii.

For anthropometric measurements, participants wore light clothes and were barefoot. We performed only non-invasive, standard anthropometric measurements. Before directly measuring body height and mass, participants self-reported these values to one of the investigators. Height and lengths were measured to the nearest millimeter using a ADE MZ10042 anthropometer (ADE Germany GmbH, Hamburg, Germany). Body mass was measured to the nearest 100 grams with a portable Tanita BWB-800P electronic scale (Arlington Heights, IL, USA). We calibrated the electronic scale for accuracy every time it was moved. Anthropometric breadths, skinfolds and circumferences were all measured on the right side of the body. Breadths were measured to the nearest millimeter using RealMet small and large sliding anthropometers (RealMet Institute, Barcelona, Spain). Skinfolds were measured to the nearest millimeter using Harpenden fat calipers (John Bull British Indicators Ltd., London, UK), with three measurements taken at each site. Circumferences were measured once to the nearest millimeter using a Cescorf anthropometric tape (Cescorf Equipamentos, Tristeza—Porto Alegre, Brazil).

#### 2.6.3 Physiological indicators

In addition to anthropometric data, blood pressure was measured with automated Omron M6 blood pressure machine outfitted with pediatric cuffs (Omron Healthcare Co., Ltd., Kyoto, Japan). Each participant was familiarized with the machine, and the appropriate cuff was selected for their arm size and adjusted where needed. The participant sat alone, quietly for at least 2 min before the measurement was performed. Resting systolic and diastolic blood pressure were measured from the participant's left arm whilst, two times at 3-min intervals.

#### 2.6.4 Other information

Parents provided information on their children's birth length, birth mass, gestational age, and breastfeeding period via a questionnaire. Girls confidentially reported the date of their first menarche on the data acquisition forms to better determine biological age at testing.

#### 2.6.5 24-h movement behavior

We assessed 24-h movement behavior (24-HMB) of youth aged 11 to 18 year using subjective and objective measures. For subjective measurements, we used a web-administered questionnaire. The physical activity section of this questionnaire was based on the School Health Action, Planning and Evaluation System (SHAPES) physical activity questionnaire (1970) (52). The original SHAPES questionnaire, a 45-multiple-choice question booklet, served as the visual template for our web-based version, maintained an identical appearance. Two key items required a 7-day recall of vigorous physical activity (VPA) and moderate physical activity (MPA), respectively. VPA was defined as “jogging, team sports, fast dancing, jump-rope, and any other physical activities that increase your heart rate and make you breathe hard and sweat.” MPA was defined as ‘lower intensity physical activities such as walking, biking to school, and recreational swimming.' Participants reported the number of hours (0–4 h) and 15-min increments (0–45 min) for each type of physical activity performed on each day of the previous week. This allowed us to collect data on intensity, duration, and frequency, enabling analyses of both weekday and weekend activity. Furthermore, we added two questions to assess compliance with physical activity recommendations for strength and bone-health exercising, also using last week's recall method.

Given the strong association between health risk of increased sedentary behavior and screen time in youth, we included four questions to describe typical screen time behavior (1. watching TV or films over the internet, 2. playing computer or video games, 3. phone activities like calling/SMS/MMS, and 4. internet browsing, including social media). These questions mirrored the format of physical activity questions, distinguishing between weekdays and weekends. Additionally, this set of questions also covered reading for pleasure (books, magazines or newsletters).

To assess sleep-related behaviors, we utilized the Pediatric Daytime Sleepiness Scale (53) augmented with questions on “in-bed' and “out-of-bed” time, for both weekdays and weekends.

Notably, the original SHAPES physical activity questionnaire has shown acceptable reliability and validity for large-scale, school-based assessments in youth (54–56). For the ACDSi 2013/14 edition, we specifically adapted the Slovenian version to integrate questions on screen time and sleep, while maintaining the original appearance to preserve its characteristics. Its proven validity and reliability within the Slovenian context (57) affirmed its suitability, leading to its integration into the My SLOfit system for collecting 24-HMB data alongside physical fitness.

To capture other aspects of 24-HMB, the questionnaire included additional items focusing on specific behaviors: strength and bone-health exercises, outdoor play, and playing musical instruments during leisure time.

To complement the 24-HMB data obtained via web-based questionnaires, we also conducted objectively measured physical activity data using the ActiGraph GTX3 accelerometers (ActiGraph, LLC, Pensacola, FL, USA) on a subsample of families. The subsample primarily included at least one child aged 10 or 11 years and one parent, and in some cases, an additional parent and/or siblings (aged between 8 and 18 years). The resulting accelerometer data was successfully collected from a total of *n* = 122 children and *n* = 123 parents/guardians. Data related to this objectively measured subsample is currently being processed in line with standard techniques.

#### 2.6.6 Commuting to school

To assess commuting behavior, the study gathered information on the modes of travel used by children aged 6–14 years to get to and from school, as well as their reasons for choosing passive transport. We employed the same commuting questions developed in the ACDSi 2013/14 edition, as their suitability for the Slovenian context has been previously established (58, 59). For children younger than 11 years, these items were completed by parents, while older children completed the questionnaire themselves. To determine home-to-school distance, parents or children either provided the exact home address or reported the distance based on Google Maps measurements.

#### 2.6.7 Organized sport activities

In addition to the standardized physical activity questions, youth aged 11 y and above were asked to disclose their current (and previous) sporting activities, including details regarding the organizational form, sport discipline, and volume of exercise performed each week.

#### 2.6.8 Motivation and self-concept

To explore the relationship between youth motivational regulations and their engagement in leisure-time physical activity, we used an adapted version of the Pictorial Motivation Scale in Physical Activity (60). Although the scale was originally designed for children with special needs, it has been successfully applied to children without disabilities (61). We also utilized the SDQ I questionnaire (62–64) to assess children's self-concepts, based on a model of academic and non-academic components of self-conception (65).

#### 2.6.9 Health status

Self-evaluation of health status was assessed in participants aged 11–18 year using the Kidscreen 10 questionnaire (66). This tool has shown strong validity and continues to be one of the most practical and effective approaches for evaluating children's overall health status.

#### 2.6.10 Family environment and close friends

Social influences on physical activity were assessed through responses provided by both youth and their parents. Family-related items captured parental characteristics (age, body height and mass) and the presence of siblings. Peer-related items focused on the child's close social circle, including the number of close friends and how many of them are physically active.

#### 2.6.11 Children's perceptions of their school environment

To assess students' perceptions of their school environment, we collected information on weekly homework loads, their most and least favorite subjects, and which subject they viewed as most important for life. Parents were also asked to provide their opinion on the latter item. Perceived school-related stress was evaluated using items targeting common stressors across several domains: environmental stressors (e.g., classroom noise, inadequate lighting), organizational stressors (e.g., early start times, short breaks), relational stressors (e.g., poor relationships with teachers or peers), academic stressors (e.g., difficulty understanding lessons, grading concerns).

#### 2.6.12 Socioeconomic environment

Previous study editions have shown lower response rates among parents from lower socioeconomic backgrounds, particularly those with limited Slovene language proficiency. To mitigate this challenge, we gathered socioeconomic information from both parents and youth. Measures included parental education level, perceived financial well-being, and family lifestyle indicators such as vacation frequency and whether the participant has a personal bedroom in the home.

#### 2.6.13 Energy drink intake

While participants were waiting to complete their anthropometric assessments, a brief interview was administered to determine the frequency and amount of energy drink and coffee intake. Data was collected on beverage types, consumption frequency, and typical volume.

## 3 Statistical considerations

### 3.1 Data treatment

All hard copy data were subject to double manual entry by the same person into a secure database to minimize transcription errors. Following the entry, descriptive statistics were computed for all variables and checked against previous ACDSi study editions to potential deviations and extreme values. Outlier detection was performed using a standardized, statistical method. In detail, the 10 most extreme outliers were manually checked. These were identified based on the largest absolute values of Studentized residuals derived from linear model that included sex, age, sex^*^age interaction, and the additional variable, which had the highest Pearson correlation within the predicted variable. Possible additional outliers (i.e., noise) and smaller outlier groups were detected using the DBSCAN clustering technique, described elsewhere (67).

Missing data handling will depend on the specific analysis. We will preferentially select analysis methods that generally allow model building in the presence of missing data. If this will not be possible, and if the proportion of missing values will be small (< 5%), we will include only participants with complete data. For a higher proportion of missing data, particularly if a sensitivity analysis will indicate a risk of bias from non-imputed data, we will perform imputation using the random forest method.

### 3.2 Generating variables for future analyses

In fitness tests results, the highest performance score will be retained for analysis, whereas in anthropometric measurements, the median of repeated values will be used to ensure accuracy and reduce measurement error. Indexes such as BMI, sum of skin folds, and other relevant metrics will be calculated according to established methods. Additionally, tests which have summative scores—such as components of motivation, self-concept, and somatotype—will be derived using their predefined protocols. The socioeconomic environment will be assessed using the FAS (68).

### 3.3 Cluster effect considerations

Given the school-based design of the ACDSi study, clustering of results within schools is possible and should be considered in data interpretation. This means that differences observed between children of a given age or constitution might be more pronounced between schools than within individual schools. To account for this potential bias in the dataset, mixed-effect models with school-effect as random factor will be employed to address this clustering effect.

### 3.4 Multilevel analyses

In addition to descriptive statistics and bivariate analyses, which will be used to characterize data distribution and identify basic associations between predictors and outcomes, multilevel multivariate analyses may be performed depending on the specific research questions addressed.

For establishing longitudinal trends Multilevel/Mixed-effects models will be used, with time (wave of a study) as a fixed effect, school clustering as a random effect (schools nested within time points) and individual-level characteristics (e.g. age, sex) as covariates. As a secondary analysis, we plan to use Age-Period-Cohort (APC) analysis to better understand the mechanism of change (cohort vs. period) and age effect.

### 3.5 Stratification of study results

Descriptive statistics will be stratified by sex and age. When testing hypotheses and constructing statistical models, analyses will generally begin with a base model for each outcome that includes sex and age (and other relevant stratification variables) as covariates. If model assumptions are violated—for example, unequal variances (heteroscedasticity) or substantially different subgroup structures—we will perform separate analyses for each sex and/or age group.

## 4 Discussion

Spanning from 1970/71 to 2023/24, the ACDSi study is among the longest-running decennial investigations worldwide focused on secular trends in child and adolescent physical fitness (see Table 6 for similar studies). It is distinguished by its comprehensive assessment of children's and adolescents' somatic and motor development, complemented by environmental indicators that help explain changes in physical fitness across sampling periods. This holistic bio-psycho-social approach allows the core research team to not only track changes in childhood physical fitness between generations, but also to observe shifts in the potential effects of various determinants of children and adolescents' physical fitness, including family, peers, the participants' socioeconomic environment, motivation, and self-concept.

**Table 6:** A comparative summary of major secular trend studies which incorporate multiple measurement timepoints and focus on physical fitness in children and adolescents.

**Name of the study**	**Sample, observing period, number of measurement time-points & region**	**Research domains**	**Variables**	**Key findings**
Secular trends in physical fitness of rural Chinese children and adolescents aged 7–18 years from 1985 to 2019 (69)	Children and adolescents 7–18 years old; *n* = 2,942,295 (1,472,655 girls) 1985–2019, 4 measurement points; CHINA	Anthropometry and physical fitness (CRF, speed, muscular strength, muscular power and flexibility).	Body weight, body height, standing long jump, 50-m dash, sit/stand-and-reach, oblique body pull-ups, pull-ups, sit-up test, 8 × 50m shuttle run, 1,000–m run, 800-m run.	BMI increased, CRF decreased throughout the years for most age and sex groups, strength increased for female and younger children, for others decrease was observed. Flexibility decreased in for male, increased for female. Power slightly increased for male, decreased for female.
Secular trends in health-related physical fitness among 11–14-year-old Croatian children and adolescents from 1999 to 2014 (70)	Children 11–14 years old; *n* = 5,077 (2,579 girls) 1999–2014, 16 measurement points; CROATIA	Anthropometry and health-related physical fitness (CRF, muscular power, coordination, muscular strength, flexibility).	Body weight, body height, standing long jump, polygon backwards, sit-up test, sit and reach, 6-min run test.	For males body size, upper-body strength and coordination increased, while flexibility, lower- body power and CRF decreased. For females body size, lower-body power, upper-body strength, coordination and flexibility increased, while CRF decreased.
Secular trends in the physical fitness of Brazilian youth: Evidence that fitness is declining for the majority but not for a fit minority (71)	Children and adolescents 6–17 years old; *n* = 65,139 (28,600 girls) 2005–2022, 17 measurement points; BRASIL	Anthropometry and physical fitness (CRF, speed, muscular strength, muscular power, agility).	Body weight, body height, 20-m dash, 6-min run test, sit-up test, standing long jump, 4 m square test, medicine ball throw.	Significant declines in physical fitness over time/year in 5 of the 6 physical fitness variables the only exception being the medicine ball throw test. Increasing variances were also observed. Fit kids are getting fitter, while less-fit kids are falling further behind.
Secular trends of cardiorespiratory fitness in children and adolescents over a 35-year period: Chronicle of a predicted foretold (72)	Children and adolescents 6–17 years old; *n* = 3,725 (1,742 girls) 1982–2017, 2 measurement points; CANADA (province of Quebec)	Anthropometry and CRF.	Body weight, body height, 20-m shuttle run test.	The results confirmed an important decrease of CRF (estimated VO2max) and functional capacity (number of shuttles) with a VO2max decrease reaching nearly −18% for males and −12% for females at the age of 17. Number of stages completed with an overall decrease of more than −30%.
Cardiorespiratory fitness has declined among French children since 1999, although the decline appears to be getting smaller (73)	Children and adolescents 6–19 years old; *n* = 15,420 (7,540 girls) 1999–2022, 15 measurement points; FRANCE	Anthropometry and CRF.	Body weight, body height, 20-m shuttle run test.	Overall decrease of CRF, but larger declines were only observed before 2010, then slight declines followed. Larger declines for unfit children but only before 2010, later slight decrease.
Temporal trends and distributional changes in cardiorespiratory fitness among Chinese children and adolescents from 1985 to 2019 (74)	Children and adolescents 7–18 years old; *n* = 1,840,212 (918,989 girls) 1985–2019, 8 measurement points; CHINA	Anthropometry and CRF.	Body weight, body height, 8 × 50-m shuttle run test, 1,000-m run/walk test, 800-m run/walk test.	Overall CRF levels of Chinese children and adolescents decreased over three decades but stabilized or improved in recent years. BMI continued to increase.
Temporal trends in the physical fitness of Hong Kong Adolescents between 1998 and 2015 (75)	Children and adolescents 12–17 years old; *n* = 28,059 1998–2015, 5 measurement points; CHINA (HONG KONG)	Anthropometry and physical fitness (CRF, muscular strength, flexibility).	Body weight, body height, 9 min run/walk test, sit-up test, push-ups, and sit-and-reach	Overall decline in CRF and trunk strength, was observed.

The ACDSi 2023/24 study experienced some recruitment challenges amongst youth aged 15–18 y, which could potentially introduce bias into the findings for this group, when comparing against previous generations. Despite this slight limitation, the ACDSi 2023/24 data collection phase has successfully continued the study's ongoing legacy, of observing physical fitness secular trends in Slovenian youth.

It is recognized that this edition's data is valuable for more than just secular trend analysis. On an international scale, it can contribute to collaborative research efforts, such as the NCD-RisC group, which researches global phenomena of non-communicable disease risk in contemporary children and adolescents, and the FitBack consortium, which focuses on promoting and establishing international comparisons of child physical fitness, specifically to advocate for greater cross-board fitness monitoring systems across Europe. This paper provides the first comprehensive description of all variables collected across all ACDSi cycles, offering researchers a deeper insight into the ACDSi data gathering and processing system. The ACDSi database, including all measurements and detailed protocol descriptions, is available to the research community for comparison and analysis upon reasonable request to the study Principal Investigators, and in compliance with GDPR regulations.

At a national level, the findings from ACDSi 2023/24, combined with the annually collected SLOfit data, will provide a clearer picture of the current state of youth physical fitness, specifically regarding the efficacy of recovery efforts following the COVID-19 pandemic. The results communicated herein have direct applications to a wide variety of professions, including pediatric practice, such as diagnostics based on nationally specific growth charts and developmental somatographs. Data from ACDSi 2023/24 will also enrich the My SLOfit online application system, which provides its users with peer- and health-related fitness feedback from additional tests like the 20-meter shuttle run and handgrip exercise, not currently covered under other surveillance programs, thereby further supporting pedagogical and sports training practices. Furthermore, the findings from this study will inform the planning and implementation of Slovenia's public health policies, such as the emerging national strategy for nutrition and physical activity currently under development.

Across the 50-year span of ACDSi data collection, substantial shifts occurred in children's daily environments (e.g., the introduction of personal computers, IT and smart TVs, smartphones, internet access, gaming platforms, and social media). To retain relevance and accurately capture contemporary behaviors, we updated questionnaire items accordingly. In different research cycles, we also included special topics that were of particular public interest at the time. These adaptations were conceptual rather than methodological—core questionnaire constructs (e.g., physical activity, sedentary behavior, sleep, self-concept, motives) remained stable, while examples and modalities were modernized to reflect technological developments and broader social changes. Whenever new behaviors emerged (e.g., smartphone screen time), new items were added following standard survey development procedures to ensure reliability and validity.

In contrast, the core measurement protocols for anthropometry and physical fitness have remained essentially unchanged since the inception of ACDSi, with the exception of a very small number of fitness tests and anthropometric measures that were discontinued or replaced when scientifically justified—for example, to improve comparability with other studies or to enable the calculation of newly developed indices and indicators such as percentage body fat derived from skinfolds. Apart from these rare adjustments, the same body measurements, fitness tests, and standardized procedures were applied consistently across all survey waves. This continuity represents a major strength of the dataset, providing exceptional comparability across decades and enabling robust analyses of long-term secular trends without the confounding effects of measurement inconsistency.

Because the ACDSi study has been conducted at the same sentinel sites for more than 50 years, it offers a unique opportunity to re-engage participants from previous editions into a new study, entitled “The Journey into Adulthood”. This study will apply a citizen science approach to investigate causal links between one's physical fitness status in childhood and the fitness- and associated-health outcomes in adulthood. As an epilogue to the ACDSi 2023/24 study, in 2025, we have already begun with adult fitness measurements of the participants of previous ACDSi study editions, creating a first step toward establishing a longitudinal approach to lifelong fitness surveillance in Slovenia.

## 5 Conclusion

By completing the 2023/24 cycle, the ACDSi continues its legacy as a long-term national program monitoring physical fitness trends in Slovenian youth since 1970/71. The current edition upholds the original study's core strengths and values by employing consistent data collection methodologies and conducting these measurements in the identical school environments as in previous decades. By maintaining the same instruments and protocols for most fitness and anthropometric assessments used since the study's inception more than fifty years ago, the ACDSi provides a stable foundation for valid comparison of results across decades. At the same time, by recognizing new challenges and integrating modern methodological approaches—such as objective 24-hour movement behavior monitoring—the 2023/24 edition has strengthened the framework for future research cycles. Together, these features ensure continuity and enhance the capacity of the ACDSi to remain a unique and influential intergenerational study well into the next decennial cycle in 2033/34 and beyond.
