# Supplementary material for: The ACDSi 2023–24 study protocol: tracking 50 years of physical fitness trends and their determinants in Slovenian children and youth
Source: Front Public Health. 2026 Jan 14;13:1734046. doi: 10.3389/fpubh.2025.1734046 (PMC12847406; doi:10.3389/fpubh.2025.1734046)
Supplement: Supplementary file 1 [file Table_1.docx]

Supplementary Material

Table 1. The research team of the 2023-24 study cycle

| Research team members, their affiliations and their role in the study | |
| --- | --- |
| University of Ljubljana, Faculty of Sport   - Prof. Gregor Jurak, Ph.D., principal investigator, study manager - Prof. Gregor Starc, Ph.D., principal investigator - Prof. Marjeta Kovač, Ph.D., investigator - Assist. Prof. Vedrana Sember, Ph.D., investigator - Assist. Prof. Neja Markelj, Ph.D., investigator - Assist. Prof. Jerneja Premelč, Ph.D., investigator - Assist. Prof. Bojan Leskošek, Ph.D., investigator - Assist. Kaja Meh, MA, investigator - Assist. Sara Besal, M.Sc., investigator - Assist. Žan Luca Potočnik, M.Sc., investigator - Špela Mikeln, M.Sc., investigator - Tjaša Rojko, M.Sc., investigator - Nika Bezjak, M.Sc., investigator - Urška Kereži, MA, investigator - Nadja Černe, student investigator - Tjaša Uljan, student investigator - Tilen Tehovnik, student investigator - Lana Vehovec, student investigator - Maks Udir, student investigator - Sara Strmec, student investigator - Brina Stropnik, student investigator - Žiga Šintler, student investigator - Klemen Ribaš, student investigator - Tinkara Dolanc, student investigator - Anja Klinc, student investigator - Nana Mihelčič, student investigator - Ana Šalamun, student investigator - Tomaž Hudales, student investigator - Kaja Gimpelj, student investigator - Jernej Muženič, student investigator - Zala Serženta Seljak, student investigator - Špela Pavlin, student investigator - Diana Kranjc, student investigator - Ivana Gadnik, student investigator - Sandi Omeradžič, student investigator - Žiga Bratanič, student investigator - Jaš Brložnik, student investigator - Vid Ključevšek, student investigator - Lovrenc Mulej, student investigator - Ula Pintar, student investigator - Nina Čulum, student investigator - Lana Vehovec, student investigator - Aneja Ljubič, student investigator - Nina Kovšca, student investigator - Ludmila Ailen Bruno, student investigator - Izabela Lužnik, student investigator - Nastja Simčič, student investigator - Daša Dolenšek, student investigator | University of Ljubljana, Faculty of Arts   - Mateja Ceglar, student investigator - Vita Jugovar, student investigator - Lučka Debelak, student investigator   University of Zagreb, Faculty of Kinesiology   - Assoc. Prof. Maroje Sorić, Ph.D., M.D., investigator   University of Ljubljana, Biotechnical Faculty   - Prof. Petra Golja, Ph.D., head of anthropometrics - Assist. Katja Zdešar Kotnik, Ph.D., investigator - Assist. Tatjana Robič Pikel, Ph.D., investigator - Nina Češnovar, student investigator - Nina Jagodic Bašič, student investigator - Petra Kaštrun, student investigator - Zala Klinar, student investigator - Gal Tom Kobe, student investigator - Jerneja Krampelj, student investigator - Karin Martinšek, student investigator - Ema Mujakić, student investigator - Kaja Pavlin, student investigator - Anja Rebernik, student investigator - Sara Sedej, student investigator - Eva Šart, student investigator - Meta Štular, student investigator - Tjaša Švigelj, student investigator - Sara Vatovec, student investigator - Kaja Benedik, student investigator - Anita Bogataj, student investigator - Anita Černevšek, student investigator - Brina Nemanič, student investigator - Anara Nemanič, student investigator - Vid Potočnik, student investigator - Špela Ručman, student investigator - Tomy Weiss, student investigator - Keti Vinko Marčec, student investigator   University of Ljubljana, Faculty of Education   - Rimi Pavlovič, M.Sc., student investigator |
| School coordinators |  |
| - Mirjam Vizjak Černe (primary school Prule, Ljubljana) - Romana Bučinel (primary school Toneta Čufarja, Ljubljana) - Simona Jurčec (primary school Ormož) - Niki Antolović Seyfert (primary school Vojke Šmuc, Izola) - Mitja Turnšek (1^st^ primary school Žalec) - Aleksandra Gričar (primary school Trebnje) - Alida Malešič Orlič (primary school Metlika) - Matjaž Gale (primary school Prežihovega Voranca, Jesenice) - Tadeja Zorč Čarga (primary school Franceta Bevka, Tolmin) - Damjan Rajh (primary school Trbovlje) - Gregor Čuk (primary school Prežihovega Voranca, Ravne na Koroškem) | - Leda Čebulj (Secondary school of commerce and design Ljubljana; technical and vocational schools) - Larisa Simončič (Secondary school of gastronomy and tourism, Ljubljana; technical and vocational schools) - Franc Bohinc (Grammar school Šiška) - Primož Praprotnik (Grammar school Šentvid) - Monika Morato (Biotechnical educational centre Ljubljana – grammar, vocational and technical schools) - Tina Oberstar (School centre Postojna; grammar school and several technical and vocational schools) - Milenko Potočnik (School centre Ptuj; several technical and vocational schools) - David Hameršak (Grammar school Ptuj) |

Table 2. Items included in the ACDSi database through editions

|  | 1983 | 1993 | 1994 | 2003 | 2004 | 2013 | 2014 | 2023 | 2024 |
| --- | --- | --- | --- | --- | --- | --- | --- | --- | --- |
| **Physical fitness tests** | ● | ● | ● | ● | ● | ● | ● | ● | ● |
| 20-s plate tapping test | ● | ● | ● | ● | ● | ● | ● | ● | ● |
| Standing broad jump | ● | ● | ● | ● | ● | ● | ● | ● | ● |
| 20-s sit-ups test (Grade 1-5) | ○ | ● | ● | ● | ● | ● | ● | ● |  |
| 60-s sit-up test |  | ● | ● | ● | ● | ● | ● | ● | ● |
| Polygon backwards test |  | ● | ● | ● | ● | ● | ● | ● | ● |
| Sit and reach test |  | ● | ● | ● | ● | ● | ● | ● | ● |
| Shoulder circumduction test |  | ● | ● | ● | ● | ● | ● | ● | ● |
| 30-s drumming test |  | ● | ● | ● | ● | ● | ● | ● | ● |
| Flamingo balance test |  | ● | ● | ● | ● | ● | ● | ● | ● |
| Flexed arm hang test | ● | ● | ● | ● | ● | ● | ● | ● | ● |
| Handgrip strength test |  |  |  |  |  | ● | ● | ● | ● |
| 30-m sprint test (Grade 1-5) | ● | ● |  | ● |  | ● | ● | ● | ● |
| 60-m dash | ● | ● | ● | ● | ● | ● | ● | ● | ● |
| 600-m run test | ● | ● | ● | ● | ● | ● | ● |  |  |
| 20-m shuttle run test [time] |  | ● | ● | ● | ● | ● | ● | ● | ● |
| 30-s plate tapping test |  | ● | ● |  |  |  |  |  |  |
| 1–foot tapping | ● | ● | ● |  |  |  |  |  |  |
| Hand and feet drumming | ● | ● | ● |  |  |  |  |  |  |
| Leg weight thrust |  | ● | ● |  |  |  |  |  |  |
| Floor dexterity |  | ● | ● | ● |  |  |  |  |  |
| Single leg balance test |  |  |  | ● | ● |  |  |  |  |
| 50-m dash | ● | ● | ● | ● |  |  |  |  |  |
| Bench sit up test | ● | ● | ● | ● |  |  |  |  |  |
| Vaulting box sit up test | ● |  |  |  |  |  |  |  |  |
| Three-plane plate tapping test | ● |  |  |  |  |  |  |  |  |
| Frontal split | ● |  |  |  |  |  |  |  |  |
| Figure of eight with low obstacle | ● |  |  |  |  |  |  |  |  |
| Running, rolling, crawling | ● |  |  |  |  |  |  |  |  |
| Running around three stands | ● |  |  |  |  |  |  |  |  |
| 1200-m run | ● |  |  |  |  |  |  |  |  |
| Balancing forward | ● |  |  |  |  |  |  |  |  |
| Single leg balancing on bench | ● |  |  |  |  |  |  |  |  |
| Walking on beam | ● |  |  |  |  |  |  |  |  |
| Match juggling | ● |  |  |  |  |  |  |  |  |
| Racket ball bounces | ● |  |  |  |  |  |  |  |  |
| Ball amortisation | ● |  |  |  |  |  |  |  |  |
| **Anthropometric measurements** | ● | ● | ● | ● | ● | ● | ● | ● | ● |
| Self-reported height [cm] |  |  |  |  |  | ● | ● | ● | ● |
| Self-reported weight [kg] |  |  |  |  |  | ● | ● | ● | ● |
| Height [cm] (parents reported) |  |  |  |  |  | ● | ● |  |  |
| Weight [kg] (parents reported) |  |  |  |  |  | ● | ● |  |  |
| Height [cm] | ● | ● | ● | ● | ● | ● | ● | ● | ● |
| Weight [kg] | ● | ● | ● | ● | ● | ● | ● | ● | ● |
| Elbow breadth (biepicondylar humerus) [cm] |  |  |  |  |  | ● | ● | ● | ● |
| Wrist breadth (lateral-medial stylion) [cm] | ● | ● | ● | ● | ● | ● | ● | ● | ● |
| Calf circumference (the widest part of calf) [cm] |  |  |  |  |  | ● | ● | ● | ● |
| Gluteal thigh circumference [cm] | ● | ● | ● | ● | ● | ● | ● |  |  |
| Mid-thigh circumference [cm] |  |  |  |  |  | ● | ● | ● | ● |
| Arm length (acromion-dactylion) [cm] | ● | ● | ● | ● | ● | ● | ● | ● | ● |
| Leg length (iliospinale) [cm] | ● | ● | ● | ● | ● | ● | ● | ● | ● |
| Foot length [cm] | ● |  |  |  |  | ● | ● | ● | ● |
| Sitting height [cm] | ● |  |  |  |  | ● | ● | ● | ● |
| Shoulder breadth (biacromial) [cm] | ● | ● | ● | ● | ● | ● | ● | ● | ● |
| Hip breadth (trochanteric) [cm] | ● | ● | ● | ● | ● | ● | ● | ● | ● |
| Pelvis breadth (biiliocristal and bispinal) [cm] |  |  |  |  |  | ● | ● |  |  |
| Femoral breadth (biepicondylar femur) [cm] | ● | ● | ● | ● | ● | ● | ● | ● | ● |
| Ankle breadth (bimalleolar) [cm] | ● | ● | ● | ● | ● | ● | ● | ● | ● |
| Triceps skinfold [mm] | ● | ● | ● | ● | ● | ● | ● | ● | ● |
| Biceps skinfold [mm] |  | ● | ● | ● | ● | ● | ● | ● | ● |
| Abdominal skinfold [mm] | ● | ● | ● | ● | ● |  |  | ● | ● |
| Suprailiac skinfold [mm] |  |  |  |  |  | ● | ● | ● | ● |
| Supraspinal skinfold [mm] |  |  |  |  |  | ● | ● | ● | ● |
| Subscapular skinfold [mm] | ● | ● | ● | ● | ● | ● | ● | ● | ● |
| Anterior thigh skinfold [mm] | ● | ● | ● | ● | ● | ● | ● | ● | ● |
| Medial calf skinfold [mm] |  |  |  |  |  | ● | ● | ● | ● |
| Mid-upper arm circumference relaxed [cm] |  |  |  |  |  | ● | ● | ● | ● |
| Mid-upper arm circumference flex ed [cm] |  |  |  |  |  | ● | ● | ● | ● |
| Forearm circumference [cm] | ● | ● | ● | ● | ● | ● | ● | ● | ● |
| Waist circumference (illiac crest) [cm] | ● |  |  |  |  | ● | ● | ● | ● |
| Hip circumference (the widest part of hips) [cm] |  |  |  |  |  | ● | ● | ● | ● |
| Endomorphic |  |  |  |  |  | ● | ● | ● | ● |
| Mesomorphic |  |  |  |  |  | ● | ● | ● | ● |
| Ectomorphic |  |  |  |  |  | ● | ● | ● | ● |
| Somatotype |  |  |  |  |  | ● | ● | ● | ● |
| Maturity offset | ● |  |  |  |  | ● | ● | ● | ● |
| Age at maturity offset | ● |  |  |  |  | ● | ● | ● | ● |
| Body mass index | ● | ● | ● | ● | ● | ● | ● | ● | ● |
| Fat mass |  |  |  | ● | ● |  |  |  |  |
| Foot phalanx circumference (left and right) [mm] | ● |  |  | ● | ● |  |  |  |  |
| Instep circumference (left and right) [mm]^1^ | ● |  |  | ● | ● |  |  |  |  |
| Heel circumference (left and right) [mm] |  |  |  | ● | ● |  |  |  |  |
| Spine deviation (thoracic and lumbar) |  |  |  |  | ● |  |  |  |  |
| Spine length (thoracic and lumbar) [mm] |  |  |  |  | ● |  |  |  |  |
| Body impedance |  |  |  | ● | ● |  |  |  |  |
| Body posture |  | ● |  | ● |  |  |  |  |  |
| Chest circumference [cm] | ● |  |  |  |  |  |  |  |  |
| Sleeve length [cm] | ● |  |  |  |  |  |  |  |  |
| Back length of torso [cm] | ● |  |  |  |  |  |  |  |  |
| Inseam length [cm] | ● |  |  |  |  |  |  |  |  |
| Head circumference [cm] | ● |  |  |  |  |  |  |  |  |
| Neck circumference [cm] | ● |  |  |  |  |  |  |  |  |
| Flat foot (Clarke angle) |  | ● | ● | ● | ● |  |  | ● | ● |
| **Physiological indicators** | ● | ● | ● | ● | ● | ● | ● | ● | ● |
| Menarche |  |  |  |  |  | ● | ● | ● | ● |
| Blood pressure [mmhg] |  |  |  |  |  | ● | ● | ● | ● |
| Resting heart rate |  |  |  | ● | ● | ● | ● |  |  |
| 20-m shuttle run test - resting heart rate |  |  |  | ● | ● | ● | ● |  |  |
| 20-m shuttle run test - average heart rate |  |  |  | ● | ● | ● | ● |  |  |
| 20-m shuttle run test - maximal heart rate |  |  |  | ● | ● | ● | ● |  |  |
| 20-m shuttle run test - minimal heart rate |  |  |  | ● | ● | ● | ● |  |  |
| Hormone therapy |  |  |  |  |  | ● | ● |  |  |
| Injuries |  |  |  |  |  |  | ● |  |  |
| Maximal vital capacity | ● | ● | ● | ● | ● |  |  |  |  |
| Forced vital capacity |  |  |  | ● | ● |  |  |  |  |
| Body hair- biological age |  | ● | ● |  |  |  |  |  |  |
| 600m run - resting heart rate |  |  |  | ● | ● |  |  |  |  |
| 600m run - average heart rate |  |  |  | ● | ● |  |  |  |  |
| 600m run - maximal heart rate |  |  |  | ● | ● |  |  |  |  |
| 600m run - heart rate 3 min after activity |  |  |  | ● | ● |  |  |  |  |
| Sport knowledge | ● | ● | ● | ● |  |  |  |  |  |
| **Birth data of a child** |  |  |  |  |  | ● | ● | ● | ● |
| Birth length [cm] |  |  |  |  |  | ● | ● | ● | ● |
| Birth mass [g] |  |  |  |  |  | ● | ● | ● | ● |
| Duration of breastfeeding [months] |  |  |  |  |  | ● | ● | ● |  |
| Gestational term status |  |  |  |  |  |  |  |  | ● |
| **24 HMB** | ○ | ○ | ○ | ○ | ○ | ● | ● | ● | ● |
| Physical activity [time]^1^ | ○ | ○ | ○ | ○ | ○ |  |  |  |  |
| MPA [min] |  |  |  |  |  | ● | ● | ● | ● |
| VPA [min] |  |  |  |  |  | ● | ● | ● | ● |
| Screen time [min] |  |  |  |  |  | ● | ● | ● | ● |
| Sleep [h] |  |  |  |  |  | ● | ● | ● | ● |
| Strength exercises (frequency) |  |  |  |  |  | ● | ● | ● | ● |
| Bone-health exercises (frequency) |  |  |  |  |  |  |  | ● | ● |
| Flexibility exercises (frequency) |  |  |  |  |  | ● | ● |  |  |
| Endurance exercises (frequency) |  |  |  |  |  | ● | ● |  |  |
| Quality of sleep |  |  |  |  |  | ● | ● | ● | ● |
| Playing an instrument [time] |  |  |  |  |  | ● | ● | ● |  |
| Spending time on the playground |  |  |  |  |  | ● | ● | ● |  |
| **Commuting to school** |  |  |  | ● | ● | ● |  | ● |  |
| Physically active arrival |  |  |  | ● | ● | ● |  | ● |  |
| Physically active departure |  |  |  | ● | ● | ● |  | ● |  |
| Mode of commuting to school |  |  |  |  |  | ● |  | ● |  |
| Distance from place of residence to school |  |  |  |  |  | ● |  | ● |  |
| Reasons to use mode of commuting to school (self-report) |  |  |  |  |  | ● |  | ● |  |
| Reasons to use mode of commuting to school (parents) |  |  |  |  |  | ● |  | ● |  |
| Sporting activity |  |  |  | ○ |  | ● | ● | ● | ● |
| Self-assessed sporting activity |  |  |  |  |  | ● | ● |  |  |
| School based extra-curricular sport activities |  |  |  |  |  | ● |  |  |  |
| School sport competitions |  |  |  |  |  | ● |  |  |  |
| Organisational form of sporting activity |  |  |  |  |  | ● | ● | ● | ● |
| Sport discipline |  |  |  |  |  | ● | ● | ● | ● |
| Weekly time spent exercising outside of school |  |  |  |  |  | ● | ● | ● | ● |
| Weekly time spent exercising in the sport club |  |  |  |  |  | ● | ● | ● | ● |
| Organisational form of sporting activity (past) |  |  |  |  |  | ● | ● | ● | ● |
| Sport discipline (past) |  |  |  |  |  | ● | ● | ● | ● |
| Years of practicing (past) |  |  |  |  |  | ● | ● | ● | ● |
| **Motivation and self-concept** | ● | ● | ● |  |  | ● | ● | ● | ● |
| Neuroticism (HANES-I and HANES-II)^1^ | ● | ● |  |  |  |  |  |  |  |
| Extraversion (HANES-I)^1^ | ● | ● |  |  |  |  |  |  |  |
| Fluency (TTCT)^1^ |  | ● | ● |  |  |  |  |  |  |
| Flexibility (TTCT)^1^ |  | ● | ● |  |  |  |  |  |  |
| Originality (TTCT)^1^ |  | ● | ● |  |  |  |  |  |  |
| Elaboration (TTCT)^1^ |  | ● | ● |  |  |  |  |  |  |
| Verbal aggression (BDHI)^1^ |  | ● | ● |  |  |  |  |  |  |
| Physical aggression (BDHI)^1^ |  | ● | ● |  |  |  |  |  |  |
| Anger (BDHI)^1^ |  | ● | ● |  |  |  |  |  |  |
| Neuroticism (EPQ)^1^ |  | ● | ● |  |  |  |  |  |  |
| Psychoticism (EPQ)^1^ |  | ● | ● |  |  |  |  |  |  |
| Extraversion (EPQ)^1^ |  | ● | ● |  |  |  |  |  |  |
| Strengths and Difficulties Questionnaire (SDQ) (Goodman) |  |  |  |  |  | ● | ● |  |  |
| Emotional symptoms (SDQ) |  |  |  |  |  | ● | ● |  |  |
| Conduct problems (SDQ) |  |  |  |  |  | ● | ● |  |  |
| Hyperactivity/inattention (SDQ) |  |  |  |  |  | ● | ● |  |  |
| Peer relationship problems (SDQ) |  |  |  |  |  | ● | ● |  |  |
| Prosocial behaviour (SDQ) |  |  |  |  |  | ● | ● |  |  |
| Barriers to physical activity |  |  |  |  |  | ● | ● |  |  |
| Intrinsic motivation (Pictorial motivation scale in physical activity) |  |  |  |  |  | ● |  | ● | ● |
| Self-determined extrinsic motivation (Pictorial motivation scale in physical activity) |  |  |  |  |  | ● |  | ● | ● |
| Non self-determined extrinsic motivation (Pictorial motivation scale in physical activity) |  |  |  |  |  | ● |  | ● | ● |
| Amotivation (Pictorial motivation scale in physical activity) |  |  |  |  |  | ● |  | ● | ● |
| Physical Ability (SDQI) |  |  |  |  |  | ● |  | ● | ● |
| Physical Appearance (SDQI) |  |  |  |  |  | ● |  | ● | ● |
| Peer Relations (SDQI) |  |  |  |  |  | ● |  | ● | ● |
| Parent Relations (SDQI) |  |  |  |  |  | ● |  | ● | ● |
| Reading (SDQI) |  |  |  |  |  | ● |  | ● | ● |
| Mathematics (SDQI) |  |  |  |  |  | ● |  | ● | ● |
| General-School (SDQI) |  |  |  |  |  | ● |  | ● | ● |
| General-Self (SDQI) |  |  |  |  |  | ● |  | ● | ● |
| Physical abilities (SDQII) |  |  |  |  |  |  |  | ● |  |
| Physical appearance (SDQII) |  |  |  |  |  |  |  | ● |  |
| Opposite-sex relationships (SDQII) |  |  |  |  |  |  |  | ● |  |
| Same-sex relationships (SDQII) |  |  |  |  |  |  |  | ● |  |
| Parent relationships (SDQII) |  |  |  |  |  |  |  | ● |  |
| Honesty-trustworthy (SDQII) |  |  |  |  |  |  |  | ● |  |
| Emotional stability (SDQII) |  |  |  |  |  |  |  | ● |  |
| Self-esteem (SDQII) |  |  |  |  |  |  |  | ● |  |
| Math (SDQII) |  |  |  |  |  |  |  | ● |  |
| Verbal (SDQII) |  |  |  |  |  |  |  | ● |  |
| General school (SDQII) |  |  |  |  |  |  |  | ● |  |
| Exercise motivation score (EMI-2) |  |  |  |  |  |  |  | ● |  |
| **Health status** | ○ | ○ | ○ | ○ | ○ | ● | ● | ● | ● |
| Long-term illness or disability |  |  |  |  |  | ● | ● | ● | ● |
| Health indicator (self-reported)^1^ | ○ | ○ | ○ | ○ | ○ | ● | ● | ● | ● |
| Health index (Kidscreen-10) |  |  |  |  |  | ● | ● | ● | ● |
| **Diet**^1^ | ○ | ○ | ○ | ○ | ○ | ● | ● | ● | ● |
| Nutritional status of child (self-assessed) |  |  |  |  |  | ● | ● |  |  |
| Nutritional status of child (parent reported) |  |  |  |  |  | ● | ● |  |  |
| Intention to change body mass |  |  |  |  |  | ● | ● |  |  |
| Frequency of consuming breakfast |  |  |  |  |  | ● | ● |  |  |
| Frequency of consuming morning snack |  |  |  |  |  | ● | ● |  |  |
| Frequency of consuming lunch |  |  |  |  |  | ● | ● |  |  |
| Frequency of consuming afternoon snack |  |  |  |  |  | ● | ● |  |  |
| Frequency of consuming diner |  |  |  |  |  | ● | ● |  |  |
| Frequency of consuming fried dishes |  |  |  |  |  | ● | ● |  |  |
| Frequency of consuming ready-to-eat main meals |  |  |  |  |  | ● | ● |  |  |
| Frequency of consuming fast food |  |  |  |  |  | ● | ● |  |  |
| Frequency of consuming ready-made and semi-prepared meals |  |  |  |  |  | ● | ● |  |  |
| Frequency of consuming whole grain bread |  |  |  |  |  | ● | ● |  |  |
| Frequency of consuming chocolate |  |  |  |  |  | ● | ● |  |  |
| Frequency of consuming beans |  |  |  |  |  | ● | ● |  |  |
| Frequency of consuming sugar sweetened beverages |  |  |  |  |  | ● | ● |  |  |
| Frequency of consuming energy drinks |  |  |  |  |  | ● | ● |  |  |
| Frequency of consuming fruits |  |  |  |  |  | ● | ● |  |  |
| Frequency of consuming vegetables |  |  |  |  |  | ● | ● |  |  |
| Eating everything on the plate |  |  |  |  |  | ● | ● |  |  |
| Availability of fruits at home |  |  |  |  |  | ● | ● |  |  |
| Availability of vegetables at home |  |  |  |  |  | ● | ● |  |  |
| Dietary supplements |  |  |  |  |  | ● |  |  |  |
| Trying tobacco |  |  |  |  |  | ● | ● |  |  |
| Frequency of smoking^1^ |  |  |  | ○ | ○ | ● | ● |  |  |
| Frequency of consuming vitamins |  |  |  |  |  |  | ● |  | ● |
| Frequency of consuming minerals |  |  |  |  |  |  | ● |  | ● |
| Frequency of consuming multivitamins or multiminerals |  |  |  |  |  |  | ● |  | ● |
| Frequency of consuming proteins and amino acids |  |  |  |  |  |  | ● |  | ● |
| Frequency of consuming fatty acids |  |  |  |  |  |  | ● |  | ● |
| Reasons for dietary supplements consumption |  |  |  |  |  |  | ● |  |  |
| Social influence on dietary supplements consumption |  |  |  |  |  |  | ● |  |  |
| Type of diet |  |  |  |  |  |  | ● |  |  |
| Healthy Nutrition Score for Kids and Youth (HuSKY) |  |  |  |  |  |  | ● |  |  |
| Coffee consumption |  |  |  |  |  |  |  | ● | ● |
| Frequency of coffee consumption |  |  |  |  |  |  |  | ● | ● |
| Consumption of energy drinks |  |  |  |  |  |  |  | ● | ● |
| Frequency of energy drink consumption |  |  |  |  |  |  |  | ● | ● |
| Most commonly used energy drink brand |  |  |  |  |  |  |  | ● | ● |
| Alcohol consumption |  |  |  | ● | ● |  |  |  |  |
| Drugs consumption |  |  |  | ● | ● |  |  |  |  |
| **Family environment and close friends** | ○ | ○ |  | ● | ● | ● | ● | ● | ● |
| Type of family |  |  |  |  |  | ● | ● | ● | ● |
| Age of parents |  |  |  |  |  | ● | ● | ● |  |
| Body height - parents (self-reported)^1^ | ● |  |  |  |  | ● | ● | ● |  |
| Body mass- parents (self-reported)^1^ | ● |  |  |  |  | ● | ● | ● |  |
| Physical activity of parents (self-reported)^1^ |  | ○ |  |  |  | ● |  |  |  |
| Sporting activity of parents (self-reported)^1^ | ○ |  |  | ● | ● |  |  |  |  |
| Sporting activity of parents in their youth (self-reported)^1^ |  | ● |  | ● | ● |  |  |  |  |
| Physical activity of parents (child reported) |  |  |  |  |  | ● | ● |  |  |
| Encouragement for child's PA |  |  |  |  |  | ● | ● | ● | ● |
| Support for child's PA |  |  |  |  |  | ● | ● | ● | ● |
| Number of siblings in the same household |  |  |  |  |  | ● | ● | ● | ● |
| Age of siblings |  |  |  |  |  | ● | ● | ● | ● |
| Number of good friends |  |  |  |  |  | ● | ● | ● | ● |
| Number of physically active friends |  |  |  |  |  | ● | ● | ● | ● |
| Owning a dog |  |  |  |  |  | ● | ● |  |  |
| Dog's activity |  |  |  |  |  | ● | ● |  |  |
| Primary dog walker in family |  |  |  |  |  | ● | ● |  |  |
| **School perception** | ● | ● | ● | ● | ● | ● | ● | ● | ● |
| The most important school subject^1^ | ● | ● | ● | ● | ● | ● |  | ● | ● |
| The hardest school subject^1^ |  | ● | ● | ● | ● | ● | ● | ● |  |
| The most favourable school subject^1^ |  | ● | ● | ● | ● | ● | ● | ● | ● |
| The least favourable school subject^1^ |  | ● | ● | ● | ● | ● | ● | ● | ● |
| The school subject that requires the most studying^1^ |  | ● | ● | ● | ● | ● | ● | ● |  |
| Perceived school related stress level^1^ | ○ | ○ | ○ | ● | ● | ● | ● | ● |  |
| Doing homework [time] |  |  |  |  |  | ● | ● | ● | ● |
| Learning at home [time]^1^ | ○ | ○ | ○ |  | ○ |  |  |  |  |
| Math grade^2^ | ● | ● | ● | ● | ● | ● | ● | ● |  |
| Final grade in the past school year^1^ | ● | ● | ● | ● | ● |  |  |  |  |
| The most important school subject (parents) |  |  |  |  |  | ● | ● | ● |  |
| Favourable components of PE class |  |  |  |  |  | ● | ● |  |  |
| Assessment of school environment^1^ | ○ | ○ | ○ | ○ |  |  |  |  |  |
| **Socioeconomic environment** | ● | ● |  | ● | ● | ● | ● | ● | ● |
| Parents education (child reported) |  |  |  |  |  | ● | ● | ● | ● |
| Parents education (self-reported)^1^ | ● | ● |  | ● | ● | ● | ● | ● |  |
| Perceived financial well-being (child reported)^1^ | ○ |  | ○ |  |  | ● | ● | ● | ● |
| Perceived financial well-being (parent reported)^1^ | ○ | ○ |  | ○ | ○ | ● | ● | ● |  |
| Number of computers in household |  |  |  |  |  | ● | ● |  |  |
| Computer in child's room |  |  |  |  |  | ● | ● |  |  |
| Own room |  |  |  |  |  | ● | ● | ● | ● |
| Family lifestyle indicator (child-reported) |  |  |  |  |  | ● | ● | ● | ● |

Notes: ^1^ – the data was collected in the years 1983 – 2004, but the data is not available in the common dataset; ○ – data was not collected using the same instrument; ● – Data was collected using the same instruments and same protocols.
